# Comparison of Efficacy of Lidocaine and Articaine as Inferior Alveolar Nerve Blocking Agents in Patients with Symptomatic Irreversible Pulpitis: Randomized Controlled Trial

**DOI:** 10.3390/medicina59101840

**Published:** 2023-10-16

**Authors:** Sobia Hassan, Alia Ahmed, Warda Saqib, Ayman M. Abulhamael, Syed Rashid Habib, Muhammad Qasim Javed

**Affiliations:** 1 Department of Periodontology, Islamic International Dental College and Hospital, Riphah International University, Islamabad 44000, Pakistan; sobia.hassan@riphah.edu.pk; 2 Department of Operative Dentistry, Islamic International Dental College and Hospital, Riphah International University, Islamabad 44000, Pakistan; alia.ahmed@riphah.edu.pk (A.A.); wardasaqib1@icloud.com (W.S.); 3 Department of Endodontics, Faculty of Dentistry, King Abdulaziz University, P.O. Box 80209, Jeddah 21589, Saudi Arabia; amahmad4@kau.edu.sa; 4 Department of Prosthetic Dental Sciences, College of Dentistry, King Saud University, King Abdullah Road, P.O. Box 60169, Riyadh 11545, Saudi Arabia; syhabib@ksu.edu.sa; 5 Department of Conservative Dental Sciences, College of Dentistry, Qassim University, P.O. Box 1162, Buraidah 51452, Qassim, Saudi Arabia

**Keywords:** Articaine, comparative study, dentistry, endodontics, inferior alveolar nerve, Lidocaine, randomized controlled trial, tooth disease

## Abstract

*Background and Objectives*: Lidocaine Hydrochloride has been the standard choice for local anesthesia in dentistry and Articaine’s unique structure and growing popularity make it a viable alternative. Due to contradictory results in prior research and a scarcity of trials conducted in the Pakistani population, this study aims to compare the anesthetic efficacy of Lidocaine with Articaine for inferior alveolar nerve blocks in patients with symptomatic irreversible pulpitis. *Materials and Methods*: This double-blinded, randomized controlled trial included 152 patients who were selected by consecutive non-probability sampling. The participants included patients who presented with symptomatic irreversible pulpitis in mandibular posterior teeth (molars and premolars) and depicted normal apical tissue radiographically. The patients were equally and randomly divided into two groups. The control group received 2% Lidocaine Hydrochloride injections, and the experiment group received 4% Articaine Hydrochloride injections. Participants scored their pain on the HP-VAS both before and after the administration of anesthesia. A value of 54 mm or less on the scale indicated effective anesthesia. The data obtained were analyzed using SPSS. Chi-square test was applied to analyze data for statistical significance. *Results*: There was no statistically significant difference in the efficacy of the two anesthetic agents. During access cavity preparation, Lidocaine demonstrated a success rate of 93%, whereas Articaine exhibited a slightly higher success rate of 97%. During initial instrumentation, the success rates for Lidocaine and Articaine were 72% and 71%, respectively. This suggests that both Lidocaine and Articaine were effective in achieving anesthesia during the dental procedure in patients with symptomatic irreversible pulpitis, with Articaine showing a slightly better success rate, although the difference was not statistically significant. *Conclusions*: The anesthetic efficacy of Articaine is similar to that of lidocaine in subjects with symptomatic irreversible pulpitis. Hence, Articaine can serve as an alternative to Lidocaine for local anesthesia administration in dentistry.

## 1. Introduction

The significance of local anesthesia (LA) in the daily practice of a dental clinician cannot be overstated. The discovery of anesthetic agents in the late nineteenth century revolutionized dentistry, globally [1]. It enabled the alleviation of pain without inducing unconsciousness. Over the years, scientists have developed various substances that can be used as local anesthetic agents (LAA) in dentistry [2]. Among these, Lidocaine Hydrochloride stands out as the preferred choice and has established itself as the gold standard, being extensively employed by dentists worldwide [3].

In the subsequent years, other amide LAA namely Prilocaine, Bupivacaine, Mepivacaine, and Etidocaine were developed. Rusching et al. achieved another milestone in dentistry by introducing Articaine, formerly known as Carticaine [4]. What distinguishes it from other amide-based LAA is the inclusion of a thiophene ring, in contrast to the typical benzene ring, and an additional ester linkage. These unique structural features contribute to its increased lipid solubility and potency [5]. Consequently, Articaine has become the anesthetic of choice for dentists across the world, including the United States and Australia [6,7,8]. In the United Kingdom, Articaine outsold Lidocaine for the first time in 2019, which is a testament to its growing popularity [9].

Numerous clinical trials have been conducted to compare the anesthetic efficacy (AE) of 4% Articaine Hydrochloride with 2% Lidocaine Hydrochloride in dental settings [6]. Existing literature offers contradictory reports regarding the effectiveness of Articaine when compared to Lidocaine. While some studies [10,11,12,13,14,15] found statistically significant differences in anesthetic ability between the two, others did not report any significant distinction [16,17,18,19,20]. A few trials concluded that the efficacy of both agents varied with the type of tooth undergoing treatment [21,22]. The inconsistency in findings among various studies can be attributed to a range of factors, including variations in dosage, injection techniques, patient diversity, study methodologies, potential publication bias, and geographical locations. Considering the divergent outcomes observed in these clinical trials, and acknowledging that certain studies lacked sufficient sample sizes, the actual disparity in the anesthetic effectiveness between these two agents remains unclear and inconclusive.

Notably, there is a dearth of research examining their AE in patients with symptomatic irreversible pulpitis of mandibular posterior teeth within the Pakistani population. This study recognizes that genetic factors, preferences, as well as variations in tooth morphology, and ethnicity among individuals from Pakistan, have the potential to impact their response to LA. This study seeks to address the limitations observed in previous research by adopting a more comprehensive approach, which includes considerations of study design and the utilization of a larger sample size.

This study aims to provide valuable insights by investigating the difference in the anesthetic efficacy of Lidocaine and Articaine as inferior alveolar nerve blocking (IANB) agents in patients with symptomatic irreversible pulpitis specifically within the Pakistani population. The null hypothesis (H_0_) for the current study is that “Articaine and Lidocaine have similar anesthetic efficacy when administered as inferior alveolar nerve blocking agents, in subjects with symptomatic irreversible pulpitis, for mandibular posterior teeth”. The alternate hypothesis (H_A_) put forth in this study supports the notion that “the anesthetic efficacy of Articaine is better than Lidocaine as an inferior nerve blocking agent in subjects with symptomatic irreversible pulpitis for mandibular posterior teeth”.

## 2. Materials and Methods

### 2.1. Ethical Considerations

Ethical approval was awarded by the institutional ethical review board at the Faculty of Dentistry, Riphah International University, Islamabad, Pakistan (Approval no: IIDC/IRC/2020/01/012). The trial protocol was registered with the Iranian Registry of Clinical Trials (http://www.irct.ir, accessed on 24 August 2023, Identifier: 61076). The research was conducted according to the World Medical Association’s Declaration of Helsinki (2013). Informed consent was obtained from all subjects involved in the study. 

### 2.2. Trial Design

**Figure 1:**
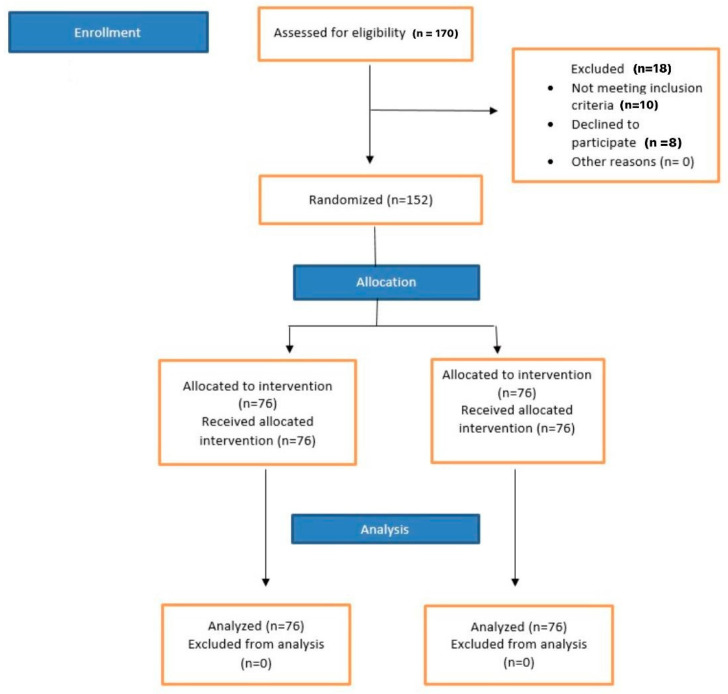
PRIRATE 2020 flowchart showing the design of the current RCT.

The design of the current randomized controlled trial (RCT) was single-centered, prospective, parallel, double-blinded, and phase III. The RCT was conducted in accordance with “The Preferred Reporting Items for Randomized Trials in Endodontics (PRIRATE)” guidelines [23]. The PRIRATE guidelines comprise a checklist that was prepared by utilizing the Clinical and Laboratory Images in Publication (CLIP), and Consolidated Standards of Reporting Trials (CONSORT) guidelines [24,25]. Figure 1 depicts the design of the current RCT.

### 2.3. Sample Size Calculation

A sample size (SS) of 152 participants was determined by utilizing the WHO SS calculator with confidence interval = 95%; significance level ≤ 0.05; study power = 80%, anticipated population proportion 1 (P1) = 0.65; and anticipated population proportion 2 (P2) = 0.45 [26]. According to this formula, a SS of 76 subjects per group was calculated. The one-tailed hypothesis was used for SS calculation. The sample was obtained using the consecutive non-probability sampling technique. 

### 2.4. Eligibility Criteria

The participants for the trial were selected at the screening clinic of the Department of Endodontics, Faculty of Dentistry, Riphah International University, Islamabad, Pakistan. The participants were recruited from 1 January to 30 June 2021, over a period of six months. The participants were fully informed about the purpose of the clinical trial and written consent (Supplementary Files: Form S1) was obtained.

#### 2.4.1. Inclusion Criteria

The trial included healthy (American Society of Anesthesiologists class I) Pakistani males and females with ages ranging from 25 to 35 years. The patients manifested symptomatic irreversible pulpitis (SIP) in mandibular premolar/molar teeth and depicted normal apical tissue, radiographically. Pulp Sensibility Testing (cold test, utilizing Endo-Ice) was conducted prior to the administration of LA to rule out pulpal necrosis. 

#### 2.4.2. Exclusion Criteria

Patients who were allergic to amides, suffering from systemic diseases, were pregnant, and those who had taken analgesics within the 24 h preceding the commencement of the endodontic procedure were not included in the RCT.

### 2.5. Randomization and Blinding

#### 2.5.1. Random Allocation SEQUENCE Generation

One hundred and fifty-two patients, who satisfied the criteria for inclusion, were randomly assigned to the control and experimental groups in 1:1. Randomization was carried out by a simple randomization technique. Online randomization software OpenEpi, (https://www.openepi.com/Random/Random.htm, last Accessed on 20 October 2020) was used for generating random allocation sequence (RAS). The total sample size (n = 152) and number of intervention arms were entered into the randomization software for RAS generation.

#### 2.5.2. Allocation Concealment

The current RCT was a double-blind trial in which both the operator and the patients were blinded to the randomized intervention that was administered. Allocation concealment, intervention preparation, and randomization were performed by an independent individual before the onset of the RCT. The investigators were blinded to the group of trial participants by securing assignment order in an opaque secured envelope. The preparation of intervention was carried out by dividing the local anesthetic medication cartridges (LAMC) into 152 (76 for each group) opaque sealed envelopes that were labeled with alphabetical symbols (A, B). Each envelope contained the LAMC, and the label of the cartridge was concealed by an opaque tap with alphabetical symbols (A or B) written on it. All LAMCs were packaged and labeled by the Septodont company and were acquired at the same time.

#### 2.5.3. Implementation

After the confirmation of SIP, the independent individual informed the single operator (SH) of which intervention should be administered. The clinician was informed about the appropriate intervention by utilizing the assigned labels (A, B) to ensure the clinician’s blinding. The researchers utilized online randomization software to assign eligible patients to either control or experimental groups (A, B). During the visit, the operator provided a standardized explanation of HP-VAS both verbally and in a written format to familiarize and train the patients in the study instrument. The patients documented the intensity of pain on HP-VAS by marking a vertical line on HP-VAS three times: pretreatment, after access cavity preparation (ACP), and after initial instrumentation. All the pre- and post-intervention research data were evaluated by a trained clinician (AA). 

### 2.6. Intervention

The patients were diagnosed with SIP based on pain history (spontaneous, and lingering pulpal pain), and confirmed by exaggerated response of the offending tooth to cold sensibility testing utilizing Endo-Ice. The offending tooth’s response to cold sensibility testing was compared to a similar unaffected contralateral tooth (Control), and the diagnosis was verified clinically by directly observing the pulpal bleeding upon access opening. Only the teeth with normal periapical tissues (verified with periapical radiographs), and negative responses to palpation and percussion tests were included in the study.

**Figure 2:**
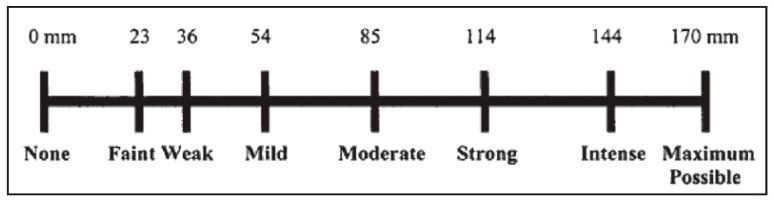
Heft–Parker Visual Analogue Scale.

The patients were assigned to the control group or experimental group, containing 76 patients each, according to the previously explained methodology. Before receiving the anesthetic injection, the patients were taught and then requested to score their pain levels using the Heft–Parker Visual Analogue Scale (HP-VAS) (Figure 2) [27]. Subsequently, IANB was administered to the participants, using either solution A or B randomly. This was followed by a waiting period of fifteen minutes to ensure adequate AE. For the IANB to be deemed successful, the lower lip needed to exhibit profound numbness, and the dental pulp needed to be adequately anesthetized during the specified timeframe. Patients failing to meet these criteria were excluded from the study [28].

Initial instrumentation (Local II) and ACP were done using a high-speed handpiece and diamond round and Endo Z burs. Successful blocks were denoted as mild to no pain on II and during endodontic ACP, scored using HP-VAS [27]. All patients completed the pre and post-injection survey. Findings were recorded in the proforma (Supplementary material: Proforma S1). 

A total of 170 patients were initially assessed for eligibility. Out of those, 18 patients were excluded: 10 did not fulfill the criteria for inclusion and 8 refused to take part in the RCT. The final participant count for the trial was 152 with 98 males and 54 females. The participants were allocated randomly, to either the control or the experimental group (n = 76 each group), to assess the efficacy of the anesthetic agents under investigation. 

#### 2.6.1. Control Group

The control group consisted of patients who received 1.7 mL of 2% Lidocaine Hydrochloride with 1:100,000 epinephrine (Septodont—Lignospan standard) and were labeled as A. 

#### 2.6.2. Experimental Group

The experimental group comprised patients who received 1.7 mL of 4% Articaine Hydrochloride with 1:100,000 epinephrine (Septodont—Septanest SP) and were labeled as B.

### 2.7. Outcome Assessment

The primary outcome assessed was the comparative efficacy of two LAA, Lidocaine and Articaine, during II and ACP. The effectiveness of LA was described as a value of <54 mm on HP-VAS (Figure 2). The HP-VAS is a 170 mm line on which several descriptive terms are mentioned. This allows the patients to subjectively mark the level of pain. HP-VAS is divided into four subcategories for the sake of data interpretation as follows [28,29]

1. No pain: 0 mm.
2. Mild pain: between 0 and 54 mm.
3. Moderate pain: between 54 and 114 mm.
4. Severe pain: ≥114 mm.

The HP-VAS, through its clear categories and descriptive language, facilitates accurate pain level assessment. 

### 2.8. Statistical Analysis

The data analysis was carried out with SPSS version 23.0. Descriptive variables (pain, and gender) were recorded as percentages and frequencies. Numerical variables (e.g., age) were documented as mean + SD. The intention to treat analysis was performed. The Shapiro–Wilk test was used to check the normality of the data. Subsequently, the AE of control and experimental groups in terms of pain control during ACP and initial instrumentation was compared by using the Chi-square test. The baseline demographic characteristics such as age and gender were also compared between the two groups using the Chi-square test. The level of significance for the Chi-square test was set at *p*-value < 0.05. Mann–Whitney U test was used to compare the mean difference in initial pain scores between control and experimental groups with a *p*-value set at <0.05.

## 3. Results

The final study sample comprised 152 patients. None of the patients were lost to follow-up. They were divided into two groups with 76 patients each: Group A (male = 48 and female = 28) was a control group that received Lidocaine. Group B (male = 50 and female = 26) was the experimental group that received Articaine. The mean age of the patients was 29.86 ± 3.36 years. There were no statistically significant variations in baseline characteristics, such as age and gender, between the two groups (*p*-value > 0.05). The difference in the mean initial pain scores of the control group (136.68 ± 27.92) and experimental group (136.45 ± 24.26) was not significant (*p*-value = 0.28).

Table 1 compares the effects of each anesthetic agent on pain scores during II and ACP between the two groups. All participants reported pronounced lip numbness 15 min after receiving an IANB. During ACP, 93% of the individuals from the control group and 97% from the experimental group were successfully anesthetized, indicating the success of the IANB. The difference between the two groups was not statistically significant. The results of our study reject the alternative hypothesis (H_A_) and support the null hypothesis (H_0_).

Furthermore, no statistically significant difference was observed between the two groups regarding the effectiveness of LA in relation to the type of tooth being treated (Table 2).

## 4. Discussion

The discovery of LAA led to the advent of pain-free dentistry. These agents have now become an indispensable part of endodontics. Without their use, routine procedures cannot be carried out optimally, primarily because of patient discomfort. The effectiveness of anesthesia, particularly the IANB, is subject to multiple factors, mainly anatomical considerations, clinical expertise, and formulation of the anesthetic agent used [30]. The current study compared the effectiveness of two anesthetic agents (Lidocaine and Articaine) as inferior alveolar nerve-blocking agents in patients with symptomatic irreversible pulpitis and normal periapical tissues. 

The present study revealed no statistically significant difference between the effectiveness of 2% Lidocaine and 4% Articaine. These results align with those of prior research carried out by Philips et al. and Simon et al. [28,31]. A similar clinical trial conducted on children under the age of 13 concluded that Articaine was as effective as Lidocaine in pediatric patients [32]. Moreover, RCT’s have reported no discernible advantage of using Articaine over Lidocaine for achieving pulpal anesthesia in mandibular teeth [33].

Although the results are statistically insignificant, Lidocaine demonstrated a 93% success rate during ACP whereas Articaine displayed a slightly higher rate of 97%. These efficacy rates are consistent with earlier studies in which the findings, while statistically insignificant, indicated a slightly better success rate for Articaine compared to Lidocaine. In an RCT on irreversible pulpitis, Brunetto and colleagues reported a 63.6% success rate for Articaine and a 54.5% success rate for Lidocaine, with no statistically significant difference in the AE of the two agents [34]. Contrary to that, in the study conducted by Tortamano et al. [26], the success rates were 65% for Articaine and 45% for Lidocaine, with a *p*-value of more than 0.05.

Certain studies have posited that Articaine may possess superior AE to Lidocaine. The trials conducted in Pakistan have compared the anesthetic effectiveness of Articaine and Lidocaine. Sheba et al. found insignificant difference between the two aforementioned agents [35]. However, Quratulain and colleagues reported that Articaine was statistically more effective during dental procedures compared to Lidocaine. This suggests that Articaine may be a viable alternative, with potentially superior AE compared to Lidocaine, in similar contexts [36]. The present study was undertaken to address the lack of research in the South Asian population, particularly in Pakistan, where existing data are limited. This study intends to supplement the preexisting body of literature on the subject within this specific region. A couple of systematic reviews comparing the AE of Articaine and Lidocaine concluded that, in dental contexts, Articaine Hydrochloride has a notable advantage over Lidocaine Hydrochloride [37]. Multiple RCTs, included in a meta-analysis, deduced that Articaine had a 1.15–2.3 times higher rate of anesthetic success compared to Lidocaine when administered as IANB [37,38,39].

Whether Articaine demonstrates anesthetic effectiveness superior to or on par with Lidocaine, it may emerge as a potential alternative to the conventionally employed Lidocaine. Although Lidocaine remains the anesthetic of choice in dentistry, and is still considered the gold standard worldwide, many reports and editorials have favored the use of Articaine. A survey conducted among dentists in New Zealand in 2016 revealed that Articaine was the most popular anesthetic agent among the majority [40]. Similarly, a study conducted among Australian dentists revealed that 70% of them used Articaine in their clinical practices [8]. However, these changing trends are not without risks. A few studies have reported incidents of paresthesia following IANB administration using Articaine [41,42]. Reportedly, the Australian Dental Association cautions against the use of Articaine for regional blocks [43]. The exact mechanism underlying paresthesia following Articaine administration remains unclear [44]. However, the literature reveals that the most probable cause for this reaction is a higher concentration of Articaine (4%) compared to conventionally used 2% Lidocaine [44,45,46]. This theory is supported by the work of Cornelius et al., when they injected 4% Articaine into the sciatic nerves of rats, it led to a loss of sensory signals in 90% of them, but with 2% Articaine, this loss was observed in only 10% of the rats [47]. These findings necessitate careful consideration and warrant further research. 

A distinctive aspect of the current research is that it compared the effectiveness of both types of anesthesia across different types of teeth, including molars and premolars. This contrasts with previous studies conducted by Henry et al. and Q Khan et al., which focused solely on the molar teeth [36,48]. Researchers have employed diverse methodologies to assess pain. Among these, the VAS has emerged as a preferred psychometric tool for pain evaluation owing to its demonstrated superiority in terms of measurement characteristics compared to discrete pain assessment scales [49]. Consequently, the VAS has gained widespread adoption within the medical research community. In our study, a modified version of the HP-VAS scale was used as previously employed by Di Renzo et al. in their pain assessment study [50]. This adapted scale features descriptive words denoting varying levels of pain, arranged along the horizontal axis in ascending order. Patients can report the intensity of their pain by marking this scale, resulting in recorded values ranging from 0 to 170, corresponding to their perceived pain intensity [51]. A value of less than 54 mm denotes effective anesthesia (no to mild pain) [28].

### 4.1. Strengths of the Study

The present research is among the few randomized controlled trials that were conducted to compare the efficacy of Lidocaine, and Articaine during ACP and root canal II, when administered as IANB in a Pakistani sub-population. Another distinctive point is that the current RCT has compared the effectiveness of both types of anesthetic agents across mandibular molar as well as premolar teeth. Therefore, despite being statistically insignificant, the results of this study offer a more extensive database for comparing and analyzing the effectiveness of anesthetic agents across various types of teeth. Furthermore, patients with asymptomatic irreversible pulpitis as well as those with pulpal necrosis were excluded from the sample to prevent the possibility of error regarding AE. 

### 4.2. Limitations of the Study

The effectiveness of anesthesia was measured using the VAS, which uses patients’ self-assessment of the extent of the pain they experience [52]. This perception varies from individual to individual owing to multiple external factors he or she may be facing. Other limitations of VAS include a ceiling effect, which hides variations in severe pain intensity, and the potential for ambiguity due to undefined anchors, limiting its sensitivity to change and leaving room for misinterpretation [53]. In addition, the study did not follow up on the subjects to rule out the possibility of any post-op sequelae, particularly paresthesia following Articaine administration, as some literature suggests. Moreover, calibration tests were not performed during the training of the patients. The subjects were restricted to a narrow age group, excluding both children and older adults. It is plausible that the results may vary when a similar investigation is carried out on a different age group and in a different setting. These limitations provide opportunities for further research initiatives. 

## 5. Conclusions

The AE of Articaine is comparable to that of Lidocaine in patients with symptomatic irreversible pulpitis. Articaine may, therefore, be a suitable alternative to Lidocaine for obtaining pulpal anesthesia during endodontic therapy, via IANB. However, it is prudent to conduct further research to determine the AE of Articaine in pediatric and geriatric patients, and to rule out the possibility of post-operative complications. 

## 6. Future Recommendations

It is recommended to conduct multi-center trials involving a study population that encompasses diverse age groups and to employ methodologies that incorporate patient follow-up. This will lead to an improved comprehension of the efficacy and possible post-injection adverse effects of different anesthetic agents. Moreover, objective measures, like Electric Pulp Testing or Cold Testing using liquid nitrogen or ethyl chloride, should be utilized for more reliable results regarding complete pulpal anesthesia. Lastly, during the training of patients, it is recommended to perform the calibration tests and report the findings in the methods. 

## Figures and Tables

**Table 1:** Comparison of anesthetic efficacy of Lidocaine vs. Articaine.

		Effectiveness of Anesthetic Injection during * N(%)	
		Access Cavity Preparation	Initial Instrumentation
Control Group (Lidocaine)	Yes	71(93)	55(72)
	No	5(7)	21(28)
Experimental Group (Articaine)	Yes	74(97)	54(71)
	No	2(3)	22(29)
*p*-value		0.246	0.857

* Measured using Heft–Parker Visual Analogue Scale—a reading of less than 54 mm denotes effective anesthesia.

**Table 2:** Comparison of Lidocaine and Articaine efficacy with respect to different tooth types.

		Lidocaine n (%)	Articaine n (%)	*p*-Value *	sig/not sig
Tooth Type	1st premolar	1 (01)	3 (04)	0.334	not sig
	2nd premolar	15 (20)	8 (11)		
	1st molar	44 (58)	49 (64)		
	2nd molar	16 (21)	16 (21)		
		76 (100)	76 (100)		

* Chi-square test.

## Data Availability

The study data is kept in password protected departmental computer as instructed by ethical committee. The data is accessible to principal investigator and statistician, only. Therefore, due to privacy concerns patients’ data can’t be shared.

## Supplementary Materials

The following supporting information can be downloaded at: https://www.mdpi.com/article/10.3390/medicina59101840/s1, Form S1: Written consent form; Proforma S1; Data collection proforma
