# Supplementary material for: Comparison of Efficacy of Lidocaine and Articaine as Inferior Alveolar Nerve Blocking Agents in Patients with Symptomatic Irreversible Pulpitis: Randomized Controlled Trial
Source: Medicina (Kaunas). 2023 Oct 16;59(10):1840. doi: 10.3390/medicina59101840 (PMC10608202; doi:10.3390/medicina59101840)
Supplement: Supplementary file 1 [file medicina-59-01840-s001.zip › medicina-2665284-supplementary.pdf]

Form S1

PATIENT CONSENT PROFORMA

ISLAMIC INTERNATIONAL DENTAL HOSPITAL

DEPARTMENT OF OPERATIVE DENTISTRY

1. It is to certify that I have been:

a) Informed thoroughly about the reasons for which my tooth needs root canal treatment

b) Informed about the potential risks and or side effects of the local anesthetics.

c) Told that this procedure is not only for the purpose of research to be conducted in the same hospital but it will also fulfill my treatment requirements

d) Informed that the confidentiality of the information will be maintained and safeguarded.

e) Satisfied by the doctor and all my queries have been answered thoroughly.

f) Assured that I am free to withdraw at any time without any comment, penalty or repercussions.

2. I also certify that the whole form has been read and explained to me in a language that I understand fully and I have agreed to participate in this study by my own free will.

Signature of the participant Signature of the Doctor

Name

Address Contact No.

Proforma S1

DATA COLLECTION PROFORMA

ISLAMIC INTERNATIONAL DENTAL HOSPITAL

DEPARTMENT OF OPERATIVE DENTISTRY

Name ________________ Age ________________ Gender ________________

Tooth (Mark x against the appropriate box):

Mandibular Right 1st Premolar		Mandibular Left 1st Premolar	
Mandibular Right 2nd Premolar		Mandibular Left 2nd Premolar	
Mandibular Right 1st Molar		Mandibular Left 1st Molar	
Mandibular Right 2nd Molar		Mandibular Left 2nd Molar	

I. Type of the anesthetic used: A ◻

B ◻

II. Level of pain experienced by the patient before administering the local anesthetic solution

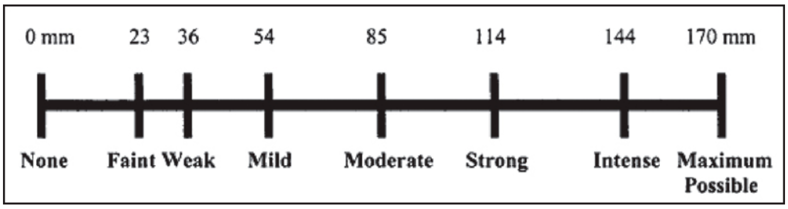

III. Level of pain experienced by the patient after administering the local anesthetic solution

IV. Signature of the patient Signature of the doctor
